# Nicotinic acid in nanocontainers. Encapsulation and release from ion exchangers

**DOI:** 10.5599/admet.626

**Published:** 2018-12-26

**Authors:** Heinrich Altshuler, Elena Ostapova, Olga Altshuler, Galina Shkurenko, Natalya Malyshenko, Sergey Lyrschikov, Roman Parshkov

**Affiliations:** 1 Laboratory of Supramolecular Polymer Chemistry, Institute of Coal Chemistry and Material Science, Federal Research Center of Coal and Coal Chemistry, Siberian Branch of Russian Academy of Sciences, Kemerovo, 650000, Russian Federation; 2 Institute of Basic Sciences, Kemerovo State University, Kemerovo, 650000, Russian Federation

**Keywords:** nicotinic acid, encapsulation, ion exchange polymers, pharmacokinetics

## Abstract

The paper is devoted to the study of the ion exchange encapsulation of nicotinic acid in nanocontainers on polymer matrices. Dowex-50 cation exchanger, sulphonated polymer based on metacyclophanoctol, polymer zirconium phosphate, and strongly basic Dowex-1 anion exchanger are used as polymer matrices. It was confirmed that commercial ion exchangers can encapsulate up to 0.64 g of nicotinic acid per gram of polymer. The high elution rate of nicotinic acid from nanocontainers via the ion exchange mechanism makes it possible to achieve the desired pharmacokinetics of drug release in vivo.

## Introduction

Currently, research is being conducted on the design of polymeric nanocontainers for drug encapsulation [1]. Polymeric nanocontainers facilitate establishment of the desired pharmacokinetics, i.e., a given drug release timeframe, decreased frequency of administration and dose, and targeted delivery of molecules to the disease site. The possibilities for preserving and storing dosage forms are practically unlimited. The therapeutic agent is used as drug delivery vector to the disease-site molecular target. Thus, the drugs encapsulation in nanocontainers creates unlimited possibilities for storing dosage forms and the change in their pharmacokinetic properties. It is important to consider the processes of encapsulation and the process of releasing substances from molecular containers immobilized on matrices of network ion exchange polymers. Dowex type ion exchangers based on polystyrene matrices are widely used in the medical industry for the preparation of drinking and pyrogen-free water, in the production of antibiotics, in a clinical setting for regulating of human water-salt balance and in the treatment of hyperkalemia by oral administrating [2-5]. Earlier we investigated the encapsulation of benzocaine and the kinetics of its desorption from sulphonated polycalixarene and CU-23 30/100 macroporous sulphocathionite [6,7]. It was suggested to use the kinetic characteristics studied to model the drug-release pharmacokinetics from a nanocontainer upon oral administration [7].

Nicotinic acid (3-pyridinecarboxylic acid, the chemical formula C_6_H_5_O_2_N, synonyms N niacin, vitamin B_3_, vitamin PP [8]) plays an important role in the human metabolism. 3-pyridinecarboxylic acid is a prosthetic group of redox coenzymes codohydrase I (nicotine adenine dinucleotide) and codohydrase II (nicotinamide adenine dinucleotide phosphate) which are key components to cellular metabolic reactions in biological systems [8,9]. Nicotinic acid as a drug is prescribed for the prevention and treatment of pellagra, with spasms of limb vessels and brain, ulcers, the neuritis of the facial nerve, infectious and gastrointestinal diseases [8]. The world demand for nicotinic acid and its derivatives continuously grows. The forecast for the year of 2020 is 100000 tons [10].

**Scheme 1.**
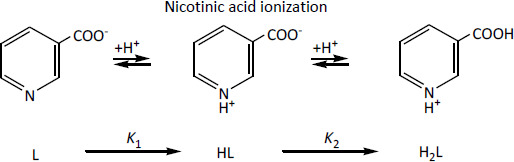
Acid-base equilibria in aqueous solutions of nicotinic acid

Free nicotinic acid is readily absorbed in all parts of gastrointestinal track. We assume that its encapsulation in ion exchangers will allow targeted delivery of the drug substance only to the stomach or intestines at oral administration. The behavior of nicotinic acid *in vivo* during passage through the gastrointestinal tract as well as by its ion exchange encapsulation and release from molecular containers is mainly determined by the acid-base equilibria (Scheme 1). See below acid-base equilibria in aqueous solutions of nicotinic acid.

**Figure 1.**
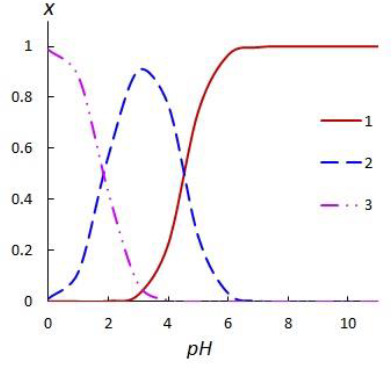
Molar fractions of ionic forms of nicotinic acid *x*_L_ (1), *x*_HL_ (2), *x*_H2L_ (3) *vs* pH of the solution.

The molar fractions of various ionic forms of nicotinic acid *vs* pH of the solution calculated from the equilibrium constants (log *K*_1_ = 4.81 and log *K*_2_ = 2.07 [11]) are shown in Figure 1.

Free hydrochloric acid is secreted in the stomach of a living organism. The pH of the medium is usually equal to 2. As can be seen from Figure 1 the concentration of nicotinic acid cations (*x*_H2L_) is predominant at pH ≤ 2. Nicotinic acid is represented by the anionic form (L) in the intestine where pH > 6. It is desirable to encapsulate and release the nicotinic acid from nanocontainer in the form of cations or anions for drug targeted delivery *in vivo*.

The aim of this paper is to study the encapsulation and the release of nicotinic acid in ionized forms proceeding in molecular containers immobilized on matrices of network ion exchange polymers.

Encapsulation of nicotinic acid in a cation form is carried out in nanocontainers on matrices of cross-linked polystyrene sulphonate (Dowex-50 cation exchanger), sulphonated polymer based on metacyclophanctol and polymer zirconium phosphate by the cation exchange reaction:


(1)

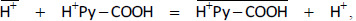



H^+^Py – COOH – cation of nicotinic acid (cation of protonated 3-pyridinecarboxylic acid, H_2_L), the line indicates the polymer phase.

Encapsulation of nicotinic acid in anion form is carried out on the matrix of cross-linked strong basic Dowex-1 anion exchanger by the anion exchange reaction


(2)

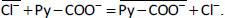



Here Py – COO^-^ – anion of nicotinic acid (anion of 3-pyridinecarboxylic acid, L).

The process (1) of the sorption of nicotinic acid cations (H_2_L) on the cation exchanger by the ion exchange mechanism has been studied at the concentration of H_2_L cations exceeding the concentration of the L anions and the HL molecules in the solution, i.e. at pH < 2 (Figure 1). The process (2) of the chloride anion exchange of by the nicotinic acid anion on the anion exchanger is studied at pH > 6, when the concentration of L anions in solution exceeded the concentration of H_2_L cations and HL molecules (Figure 1).

## Experimental

### Materials

Nicotinic acid (obtained from J.S.C. «Organica», Russian Federation) contains 99.0 % 3-pyridinecarboxylic acid and meets the requirements of the International Pharmacopoeia [12]. Dowex-50 cation exchanger and strongly basic Dowex-1 anion exchanger were purchased from Sigma-Aldrich.

Dowex-50, cation exchanging sulphonated copolymer of styrene with 8 % divinylbenzene, contains only one type of ionogenic groups, *viz*. sulpho group (SO_3_H). The total dynamic ion exchange capacity is 5.2 mequiv/g of the H-form of a dry polymer. A sulphonated polymer based on metacyclophanoctol was prepared according to the procedure described previously [13]. It has a gel structure and contains two types of ionogenic groups: phenolic -OH and -SO_3_H groups. The total dynamic ion exchange capacity of sulphonated polycalixarene is 5.65 mequiv./g. Its capacity with respect to sulpho groups is 2.45 mequiv./g [13]. Polymer zirconium phosphate is prepared according to the procedure described previously [14]. The dynamic ion exchange capacity with respect to 0.1 M NaCl is 1.05 mequiv./g.

Dowex-1 is a strong basic anion exchanger. It contains benzyltrimethylammonium groups at the matrix of the styrene with 8 % divinylbenzene copolymer. The total dynamic ion exchange capacity of the anion exchanger with respect to 0.1 М КОН is equal to 2.7 mequiv. per 1 g of the Cl-form of the dry polymer.

### Methods

The encapsulation of nicotinic acid in nanocontainers was carried out by the ion exchange sorption. To encapsulate nicotinic acid into nanocontainers on the matrices of the cation exchangers and to determine the dynamic capacity of the cation exchangers with respect to nicotinic acid, 0.01 M solution of nicotinic acid in 0.01 M hydrochloric acid was passed through an ion exchange column containing cation exchanger in H-form until the solution composition at the inlet and outlet from the column became equal. To encapsulate nicotinic acid into a nanocontainer on the anion exchanger, 0.01-0.05 M solution of the potassium salt of nicotinic acid was passed through an ion exchange column containing Dowex-1 in the Cl-form until the nicotinic acid concentration at the inlet and outlet from the column became equal. The dynamic ion exchange capacity with respect to nicotinic acid was calculated as the arithmetic mean of 7 measurements on each polymer.

To elute nicotinic acid from nanocontainers on the cation exchanger matrices, a 0.01 M HCl solution was passed through the cation exchanger layer containing nicotinic acid until the nicotinic acid in the eluate disappeared. When the nicotinic acid was eluted from the Dowex-1 anion exchanger through the ion exchanger layer containing nicotinic acid, 0.01-0.1 M aqueous NaCl solutions was passed. The nicotinic acid was crystallized from the obtained eluates by their evaporation and precipitation at pH 3.3-3.6 via the procedure [15].

The selection of spherical granules and their size determination for kinetic studies was carried out on the IMC 100 × 50, A microscope. The size distribution of polymer granules is described by the Gaussian function. The radius of spherical particles calculated as the arithmetic mean of 1000 granules sizes was equal to (1.8±1.2)·10^-4^ m for Dowex-50 cation exchanger and (2.2±0.6)·10^-4^ m for Dowex-1 anion exchanger. The errors were calculated with 0.95 confidence level. The kinetics of the nicotinic acid release (elution) from nanocontainers was studied at 298 K using the dynamic thin-layer method [16]. The fact that the solution flowed through a thin layer of the ion exchanger at high speed is a special feature of the method [16]. The infinite volume of 0.01 M aqueous HCl solution or pure water were passed through the layer of Dowex-50 cation exchanger in the form of nicotinic acid cations. The infinite volume of 0.1 M aqueous NaCl solution was passed through the layer of Dowex-1 anion exchanger in the form of nicotinic acid anions. After a certain period of time the concentrations of nicotinic acid were determined using an SF-46 spectrophotometer at λ = 262.7 nm in a buffer solution with pH 6.9. The degree of conversion was calculated using the formula *F = M*_t_*/M_∞_*, where *M*_t_ is the amount of nicotinic acid desorbed up to time *t* and *M_∞_* is the amount of nicotinic acid desorbed up to infinite time.

^13^C NMR solid-state spectra were obtained on a Bruker Avance II+ 300WB instrument at the operating frequencies 75.48 MHz (^13^C). Fourier IR spectra of nicotinic acid in tablets with KBr were obtained on the "Infralum FT-801" spectrometer.

Molecular design and calculations of the formation energies of nanocontainer structures containing encapsulated nicotinic acid were carried out by the PM7 method in the framework of the MOPAC2016 program taken from the web site: www.openmopac.net [17] on a computer based on the Intel (R) Core (TM) processor i5-2310 CPU @ 2.9 GHz 2.9 GHz.

## Results and Discussion

### Nanocontainers

**Figure 2.**
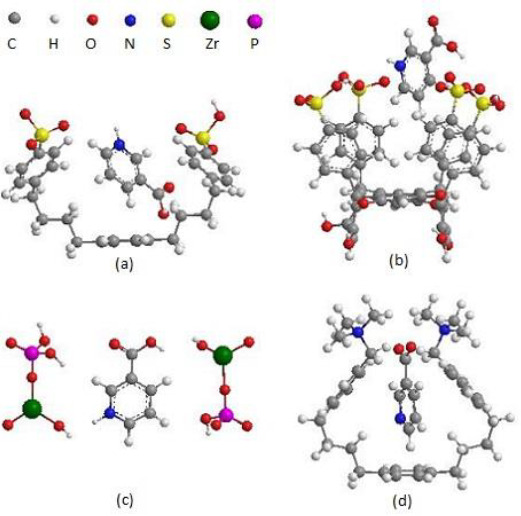
The structures of nanocontainers containing nicotinic acid in (a) Dowex-50 cation exchanger, (b) sulphonated polymer based on metacyclophanoctol, (c) polymer zirconium phosphate, (d) Dowex-1 anion exchanger. The structures have a minimum of internal energy within the MOPAC2016 program.

Elementary units of network polymers, which act as nanocontainers for nicotinic acid are shown in Figure 2.

Elementary units of Dowex-50 cation exchanger (Figure 2(a)) and Dowex-1 anion exchanger (Figure 2(d)) contain hydrophobic baskets consisting of alkylaromatic chains with ionogenic sulpho- or tetraalkylammonium group, respectively. The elementary unit of sulphonated polymer based on metacyclophanoctol (Figure 2(b)) contains a macrocyclic cavity and two hydrophilic rims. The lower rim includes eight hydroxyl groups; the upper rim consists of four ionogenic sulpho groups (one SO_3_H group per benzene ring). Polymer zirconium phosphate is a matrix with a layered structure, which is capable of locating small guest molecules in the interlayer space. The elementary unit of this polymer is shown in Figure 2(c). As shown *via* quantum-chemical calculations, the elementary units of the investigated polymers complementarily interact with ionized nicotinic acid molecules (cations of protonated 3-pyridinecarboxylic acid or anions 3-pyridinecarboxylate) by host-guest type.

### Encapsulation

The experimental values of the dynamic exchange capacity of polymers at the encapsulation of nicotinic acid are given in the Table 1.

It is found out that 0.33 and 0.64 grams of nicotinic acid are encapsulated in one gram of Dowex-1 and Dowex-50, respectively. The exchange capacities of the Dowex-50 cation exchanger and the sulphonated polymer based on metacyclophanoctol with respect to nicotinic acid (Table 1) correspond to the contents of strongly acidic sulpho groups in polymers. The capacity of the Dowex-1 anion exchanger with respect to nicotinic acid corresponds to the content of benzyltrimethylammonium ionogenic groups in the polymer.

**Figure 3.**
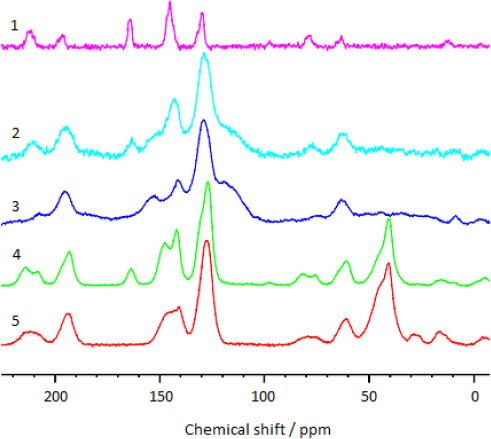
^13^C NMR spectra of solid samples of (1) nicotinic acid sulfate, (2) sulphonated polymer based on metacyclophanoctol containing nicotinic acid, (3) sulphonated polymer based on metacyclophanoctol, (4) Dowex-50 cation exchanger containing nicotinic acid, (5) Dowex-50 cation exchanger.

**Figure 4.**
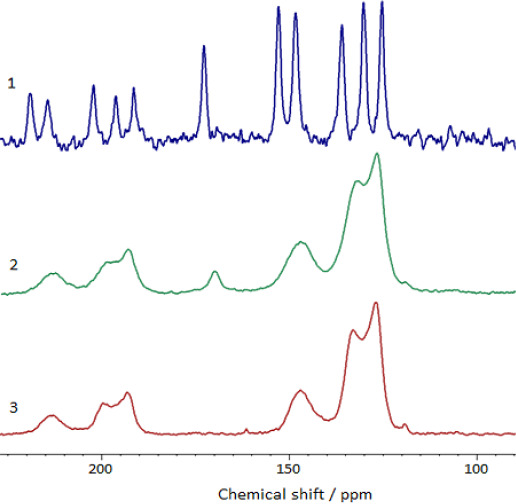
^13^C NMR spectra of solid samples of (1) of potassium salt of the nicotinic acid (potassium 3-pyridinecarboxylate); (2) Dowex-1 anion exchanger containing nicotinic acid anions; (3) Dowex-1 anion exchanger.

^13^C NMR spectra of the solid samples of nicotinic acid sulfate (sulfate of protonated 3-pyridinecarboxylic acid), sulphonated polymer based on metacyclophanoctol and Dowex-50 cation exchanger are given in Figure 3. ^13^C NMR spectra of the solid samples of the potassium salt of the nicotinic acid (potassium 3-pyridinecarboxylate), Dowex-1 anion exchanger are given in Figure 4. As can be seen from Figure 3, there is a resonance line corresponding to the chemical shift of *>* = 165 ppm (C = O [18]) in the spectrum (1) of nicotinic acid sulfate and in the spectra (2), (4) of cation exchangers containing encapsulated nicotinic acid. In the ^13^C NMR spectrum (Figure 4) of the potassium salt of the nicotinic acid as well as in the spectrum of the Dowex-1 filled with nicotinic acid anions, there is a resonance line next to *>* = 170 ppm (chemical shift of carboxylate anions [18]). There is no such line in spectrum of the Dowex-1, free of nicotinic acid. Thus, it follows from the ^13^C spectra that the encapsulated nicotinic acid is actually contained in ion exchange polymers in an ionized forms as protonated 3-pyridinecarboxylic acid cations or as anions of 3-pyridinecarboxylate.

### Release

Water solutions simulating the electrolyte composition of the human gastrointestinal tract are used for the release (elution) of the nicotinic acid from nanocontainers. The release of nicotinic acid from cation exchangers is carried out using HCl solution (at pH ≤ 2). This simulates the electrolyte composition of the stomach. The release of the nicotinic acid from the anion exchanger is carried out using aqueous solutions of NaCl (at pH > 6) which simulates the electrolyte composition of the intestine.

It has been found that the dynamic exchange capacities during encapsulation and the release of nicotinic acid are equal to each other. It is clear that the encapsulation of the nicotinic acid proceeds in accordance with the stoichiometric ion exchange reactions (1) and (2). The release of nicotinic acid from nanocontainers on matrices of ion exchange polymers can be described by the ion exchange reactions (3) and (4).


(3)

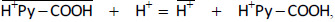




(4)

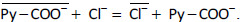



Nicotinic acid that is precipitated from eluates contains 99 % of the 3-pyridinecarboxylic acid. The melting point of 235–236 2С corresponds to these data [19]. Elemental analyses (%) of the precipitated nicotinic acid are C, 57.9 ± 0.5; H, 4.1 ± 0.1; N, 11.2 ± 0.5; O, 25.0 ± 1.0 which corresponds within the error of determination to the calculated C_7_H_5_NO_4_ formula (C, 58.30; H, 4.08; N, 11.34; O, 25.91). Thus, the composition of the product released from the encapsulated state coincides with the elemental composition of the nicotinic acid.

**Figure 5.**
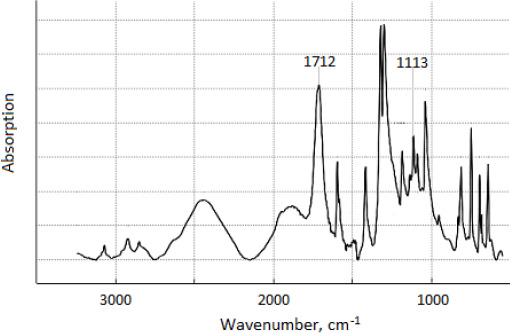
Fourier IR spectrum of nicotinic acid precipitated from eluate in tablets with KBr.

The Fourier IR spectrum of the product precipitated from the eluate corresponds (Figure 5) to the spectrum [20] of nicotinic acid. It contains the intense vibration bands at 1712 сm^– 1^ and 1113 сm^– 1^ belonging to the carbonyl and carboxyl group of the 3-pyridinecarboxylic acid [20,21]. The ^13^С NMR spectrum of the product obtained from the eluate coincided with the NMR spectrum of the nicotinic acid. The nicotinic acid is encapsulated, and then completely released from the researched polymers. This is resulted from the material balance of the ion exchange processes (1), (2) of encapsulation and (3), (4) release, NMR and IR spectra, elemental analysis, and melting point of nicotinic acid. Nicotinic acid in nanocontainers exists in the cation or anion forms.

It is established that Dowex-50 cation exchanger, sulphonated polymer based on metacyclophanoctol, polymeric zirconium phosphate as well as a strongly basic Dowex-1 anion exchanger were not destroyed, during the process of encapsulating and releasing of the nicotinic acid. The performance characteristics of commercial polymers Dowex-1 and Dowex-50 were not changed in five cycles of encapsulation and during the release of the nicotinic acid. Taking into account the above, the nicotinic acid encapsulation in nanocontainers based on commercial polymers: Dowex-50 cation exchanger and Dowex-1 anion exchanger expands the possibilities of obtaining prolonged forms of the active substance.

### Kinetics

**Figure 6.**
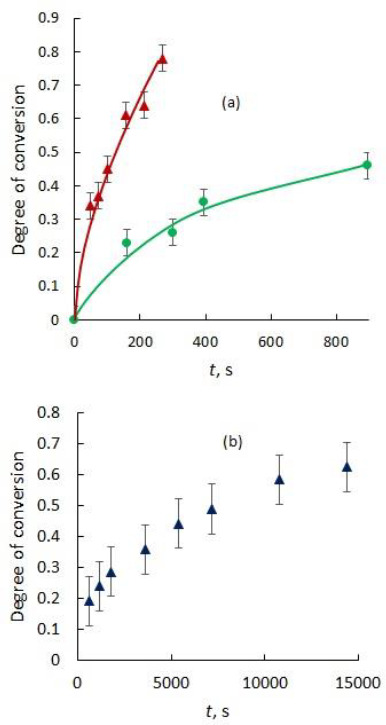
Release-time profile of nicotinic acid from ion exchangers. (a) Red triangles represent the experimental data of nicotinic acid release from Dowex-50 by 0.01 М HCl, green circles represent the experimental data of nicotinic acid release from Dowex-1 by 0.1 М NaCl. (b) Dark blue triangles represent the experimental data of nicotinic acid release from Dowex-50 by H_2_O. Error bars show deviation from mean values, n=3.

The release vs time profile of nicotinic acid from commercial ion exchangers is shown in Figure 6. As can be seen, the best nicotinic acid release profile is achieved on the Dowex 50 ion exchanger using 0.01 M HCl as eluent.

The research of the kinetics and mechanism of the ion exchange processes (3) and (4) of the nicotinic acid release from nanocontainers is of current interest. The dependences form of the conversion degree on time and the passage of lines through the origin of coordinates (Figure 6(a)) in accordance with the known criteria [22] indicate that the mechanism of the nicotinic acid release from ion exchangers using HCl or NaCl is controlled by diffusion of exchangeable ions into the polymer; i.e., gel diffusion kinetics of ion exchange occurs. The gel diffusion kinetics of ion exchange in the case of constant diffusion coefficient and spherical symmetry is described [23] by the differential equation:


(5)

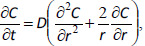



where *D* is the diffusion coefficient of the component, *C* is the current concentration of the component in the polymer, and *r* is the value of radius vector.

In a monofunctional ion exchanger, the diffusion kinetics of ion exchange in a spherical polymer particle contacting with a solution of invariable composition and infinite volume is described by the known dependence [24]:


(6)

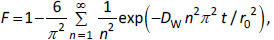



where *D*_W_ is the interdiffusion coefficient of exchangeable ions in the polymer, *r*_0_ is the radius of a polymer particle.

**Figure 7.**
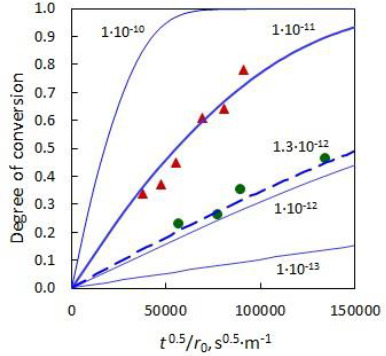
Kinetics of the processes of nicotinic acid release from ion exchangers. The solid and dotted lines correspond to calculations using Equation (6); the numbers near the lines show the interdiffusion coefficients expressed in m^2^/s. The red triangles represent the experimental data of nicotinic acid release from Dowex-50 and green circles represent the experimental data of nicotinic acid release from Dowex-1.

The kinetic characteristics of the processes (3) and (4) of nicotinic acid release are shown in Figure 7.

A comparison of the time dependences of the conversion degree and the experimental data (at 0 < *F* < 0.5) shows that the kinetics of processes (3) and (4) are determined by a slow diffusion of the components in the polymer phase. This is confirmed by the agreement between the experimental kinetic characteristics and theoretical equations as well as low values of the diffusion coefficients characteristic of the polymer phase.

As one can see from Figure 7, the experimental data on the release of the protonated nicotinic acid from polymer are approximated well by Equation (6). The interdiffusion coefficient of organic cations is (0.9 ± 0.1)·10^−11^ m^2^/s in Dowex-50 while the interdiffusion coefficient of organic anions in Dowex-1 is (1.3 ± 0.4)·10^-12^ m^2^/s. For the processes involving ionized pyridinecarboxylic acid the interdiffusion coefficients are significantly lower than the interdiffusion coefficient for the ion exchange in Amberlite IR-1 (3.7·10^-10^ m^2^/s [24]). For the processes (3) and (4), the interdiffusion coefficients within the selected interval of compositions of the ion exchangers are definitely controlled by the mobility of the organic ions in the polymers. For F < 0.5 the interdiffusion coefficient errors were calculated with a confidence equal to 0.95 using the least squares method.

The half-conversion time of nicotinic acid release is obtained from experimental data (Figure 6 ) or is calculated by equation (6) using the values of interdiffusion coefficients. The half-conversion time of nicotinic acid release by 0.01 M solution of HCl from Dowex-50 is 140 s, which equals to 10,000 s when nicotinic acid is desorbed by water. The half- conversion time of nicotinic acid release by 0.1 M solution of NaCl from Dowex-1 is 1200 s that equals to more than one year when nicotinic acid is desorbed by water.

## Conclusions

The nicotinic acid encapsulation in nanocontainers based on commercial polymers: Dowex-50 cation exchanger and Dowex-1 anion exchanger expands the possibilities of obtaining prolonged forms of the active substance.

Nicotinic acid is released from the polymers by the ion exchange mechanism using a strong electrolyte as the eluent. This should be taken into account when predicting the pharmacokinetics of the release of a drug substance *in vivo.* It is likely that nicotinic acid encapsulated in Dowex-50 upon oral administration remains immobilized in the esophagus and it is rapidly released at pH 2 upon reaching the stomach where hydrochloric acid is secreted. Nicotinic acid encapsulated in Dowex-1 can be targeted to the intestine in which it is easily released at pH 6.

## Figures and Tables

**Table 1.** Capacities of ion exchange polymers with respect to nicotinic acid (mean ± error, 0.95 confidence level).

Ion exchange polymer	Equilibrium solution	Dynamic exchange capacity (content of nicotinic acid per one gram of dry polymer), mmol/g
Dowex-50 cation exchanger	0.01 M nicotinic acid in 0.01 M HCl	5.2 ± 0.1
Sulphonated polymer based on metacyclophanoctol	0.01 M nicotinic acid in 0.01 M HCl	2.45 ± 0.05
Polymer zirconium phosphate	0.01 M nicotinic acid in 0.01 M HCl	0.18 ± 0.02
Dowex-1 anion exchanger	Potassium nicotinate in water, 0.01 M	2.70 ± 0.05
